# The value of restaging CT following neoadjuvant chemotherapy for resectable gastric cancer. A population-based study

**DOI:** 10.1186/s12957-021-02313-3

**Published:** 2021-07-13

**Authors:** Alina Desiree Sandø, Reidun Fougner, Jon Erik Grønbech, Erling Audun Bringeland

**Affiliations:** 1 Department of Gastrointestinal Surgery, St. Olavs Hospital, Trondheim University Hospital, 7006 Trondheim, Norway; 2 Department of Cancer Research and Molecular Medicine, Norwegian University of Science and Technology, Trondheim, Norway; 3 Department of Radiology St. Olavs Hospital, Trondheim University Hospital, Trondheim, Norway

**Keywords:** Gastric cancer, Response evaluation, Neoadjuvant chemotherapy, Downstaging

## Abstract

**Background:**

Response evaluation following neoadjuvant chemotherapy (NAC) in gastric cancer is debated. The aim of this study was to investigate the value of UICC-*downstaging* as mode of response evaluation following a MAGIC-style regimen of NAC.

**Methods:**

Retrospective, population-based study on consecutive patients with resectable gastric adenocarcinoma receiving NAC from 2007 to 2016. CT-scan was obtained at diagnosis (rTNM) and repeated following NAC (yrTNM) to evaluate response in terms of *downstaging*. Further, yrTNM stage was crosstabulated to pathologic stage (ypTNM) to depict correlation between radiologic and pathologic assessment.

**Results:**

Of 171 patients receiving NAC, 169 were available for response evaluation. For TNM-stages, 43% responded, 50% had stable disease and 7% progressed at CT. Crosstabulating yrTNM stage to ypTNM stage, 24% had concordant stages, with CT overstaging 38% and understaging 38% of the tumours, Cohen kappa ƙ = 0,06 (95%CI 0.004–0.12). Similar patterns of discordance were found for T-stages and N-stages separately. For M-category, restaging CT detected 12 patients with carcinomatosis, with an additional 14 diagnosed with carcinomatosis only at operation. No patient developed parenchymal or extra abdominal metastases, and none developed locally non-resectable tumour during delivery of NAC. Restaging CT with response evaluation was not able to stratify patients into groups of different long-term survival rates based on response mode.

**Conclusions:**

Routine CT-scan following NAC is of limited value. Accuracy of CT staging compared to final pathologic stage is poor, and radiologic *downstaging* as measure of response evaluation is unreliable and unable to discriminate long-term survival rates based on response mode.

## Background

Since the publication of the British MAGIC trial in 2006, perioperative chemotherapy has been standard of care in Europe for resectable gastric cancer [1–3]. The regimen was recently superseded by the German FLOT4 following a randomized controlled trail comparing the two head on, concluding with improved pathologic response and long-term survival rates for the latter [2, 4]. Evidence suggests a differential effectiveness of perioperative chemotherapy on clinicopathologic variables as stage, histological subtypes and genomic profile [2, 5–9]. For the MAGIC regimen, up to 15% experienced disease progression, even to metastatic disease, indicating that not all benefit from neoadjuvant chemotherapy (NAC) [4, 7, 10]. Still, NAC is persistently administered as a “one size fits all” treatment.

Two principally different approaches for evaluating tumour response following NAC are available. First is histologic criteria like the Becker tumour regression grade or the Mandard score [11, 12]. Second is response evaluation by standardized CT scans before and after chemotherapy, either as UICC-*downstaging* or by measuring *downsizing* with methods like RECIST (Response Evaluation Criteria in Solid Tumours) [13]. No consensus exists on the preferred method, and implications of findings are still debated [7, 14].

The aim of this study was to explore how standardized CT-scans perform when used to evaluate response following NAC in terms of UICC-*downstaging* and measure concordance between radiologic assigned stage (yrTNM) and pathologic stage (ypTNM) as a marker of the reliability of the radiologic downstaging.

## Material and methods

### Study design

**Fig. 1:**
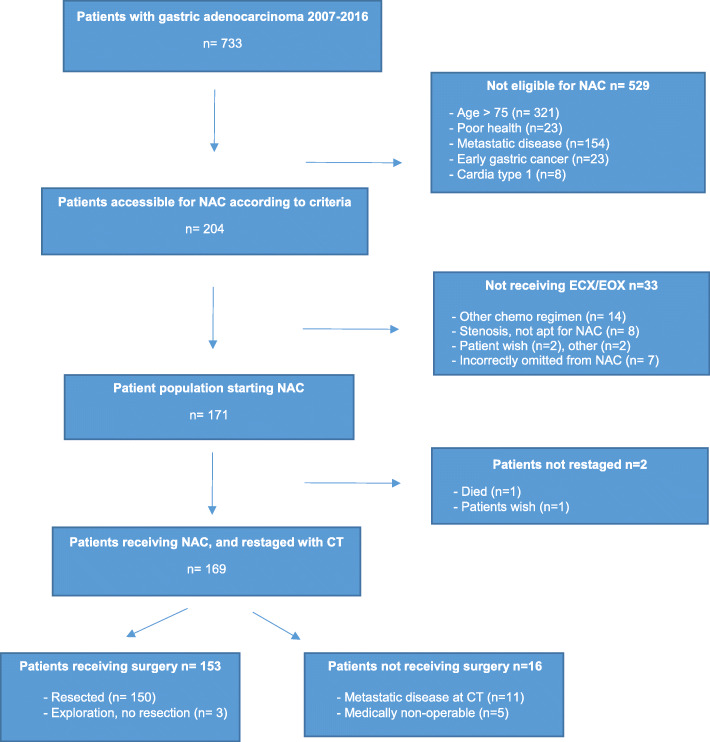
Flowchart, patient population. Study flowchart identifying the *n* = 171 patients constituting the study cohort among 733 consecutive patients diagnosed with gastric adenocarcinoma in Central Norway 2007–2016 (NAC, neoadjuvant chemotherapy)

The study design is a retrospective, population-based study from Central Norway. Since 2006, evaluation and treatment of gastric cancer was centralized to St. Olavs Hospital, the university hospital of the region, with a catchment area of 700.000, constituting some 14% of the Norwegian population. All patients with histologically verified gastric adenocarcinoma from 1 January 2007 to 31 December 2016 were identified through the *Norwegian Cancer Registry* (NCR) and *The Norwegian Patient Register* (NPR). Individual electronic patient journals (EPJ) were reviewed to secure a complete registration of relevant clinical variables for 733 patients. Patients in a curative setting with tumours of true gastric or cardia types II/III location, receiving perioperative chemotherapy, were the objective for further study. Established criteria for perioperative chemotherapy were clinical stage Ib–III, age below 75 years, and WHO 0-1 performance status, leaving 171 to constitute the study cohort (Fig. 1). The study was approved by the Regional Ethics Committee. The manuscript was prepared in accordance with the STROBE guidelines.

### Perioperative chemotherapy

As part of national standard, a MAGIC style regimen of perioperative chemotherapy was introduced in 2007 for resectable gastric cancer at St. Olavs Hospital. Oral capecitabin (xeloda®) 1250 mg/m^2^ was given for 21 days, i.v. oxaliplatin 130 mg/m^2^ or i.v. cisplatin 60 mg/m^2^ on day 1, and i.v. epirubicin 50 mg/m^2^ on day 1. The EOX/ECX regimen was delivered with three cycles prior to surgery and three to follow for radically resected patients.

### Tumour staging and response evaluation

All patients were discussed at multidisciplinary meetings following initial staging with CT and gastroscopy. In accordance with national guidelines endoscopic ultrasound, PET-CT or laparoscopy prior to NAC was not routinely used [15]. Radiologic staging was done with a multidetector CT (Siemens Somatotom Definition Flash or AS+, with detector 128 × 0.6), with repeated imaging for restaging following NAC. The procedure was standardized and the protocol designed for optimal gastric distention [16, 17]. Following a fasting period of four hours, 20 mg butylscopolamine bromide was administered i.v and 1 l of tap water orally together with effervescent granules. Intravenous contrast was given as Omnipaque 350 mg/ml at a rate of 4 ml/s, volume depending on the patient’s weight (120–180 ml). CT images were obtained after 45 and 70–75 s. Images were reconstructed to series of 1.5 and 3 mm thin slices in the axial, coronal and sagittal planes. There was no use of three-dimensional rendering.

For study purposes, a senior gastro radiologist, blinded to final ypTN stage did the radiologic response evaluation. Disease stage at baseline and following NAC were set as follows: T1, invisible tumour or focal thickening of mucosal layer; T2, focal thickening of the gastric wall with smooth outer border; T3, diffuse or focal transmural thickening of the gastric wall with blurry border to periventricular fat; and T4, tumour infiltrating the serosal lining or other organs. Lymph nodes were considered malignant when short axis enlarged to ≥ 10 mm or showing a pathologic structure. M+ category was defined as malignant peritoneal deposits, paraaortic nodes or organ metastases. Radiologic *downstaging* as a measure of response was defined as a lowering of disease stage one or more tiers at CT, disease progression as migration to a higher stage, otherwise judged as stable disease. The Union for International Cancer Control (UICC) and the American Joint Committee on Cancer (AJCC) TNM classification 7th. edition was used [18].

### Statistics

Continuous variables are reported as mean (range). Categorical variables are crosstabulated and analysed using the χ^2^ test. Concordance was defined as proportion of stages coincident at the diagonal on crosstabulation. The Cohen kappa statistic was used to determine the correlation between radiologic yrTNM and pathologic ypTNM stages. Overall survival rates were analysed using the Kaplan-Meier method and compared by the log-rank test. Level of statistical significance was set at *p* = 0.05. Analyses were done using IBM SPSS Statistics version 27.

## Results

A total of 171 patients with mean age 63 years (range 27–77) started perioperative chemotherapy (Fig. 1). ECX was given to 57 (33%) and EOX to 114 (67%). Tolerability of NAC was on par with numbers reported by others [2, 3] with 145/171 (85%) completing the three preoperative cycles and 32/171 (19%) having one or more dose reductions.

### Radiologic response evaluation: CT stage at diagnosis (rTNM) vs CT stage following NAC (yrTNM)

**Fig. 2:**
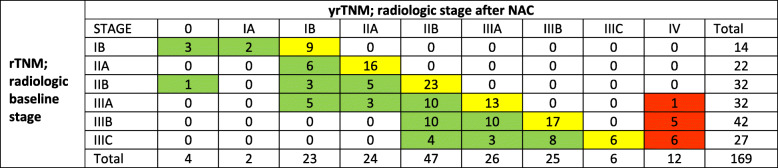
Radiologic response evaluation. Crosstabulation of CT-stage at diagnosis (rTNM) vs CT-stage following neoadjuvant chemotherapy (yrTNM), *n* = 169. Numbers on stage migration express response/progression. Green = downstaging/response, yellow = stable stage, red = upstaging/progression

Two patients were unavailable for response evaluation, one died during NAC and one refused further imaging and treatment, leaving 169 patients to compare CT stage at diagnosis (rTNM) to CT stage following NAC (yrTNM). (Table 1, Fig. 2). For radiologic UICC-stage, 73/169 patients (43%) responded, 50% had stable disease and 7% had progression (Fig. 2). Only four patients were appraised as radiological complete responders. Of the 7% with progression, all had stage III disease at the outset, all progressed to peritoneal carcinomatosis and none developed parenchymatous organ metastases. Radiologic response crosstabulated on several clinical variables showed that gender, age, Lauren type or number of NAC cycles were not significantly related to response category. Tumour location and T stage had a significant bearing on response mode, accounted for by a lack of response for tumours of anatomic diffuse location and T4 cancers, respectively (Table 1). Assessing T-stages isolated, none of the tumours migrated to a higher category at CT, 121/169 (72%) were appraised as stable and 48 (28%) migrated to a lower T-category following NAC. Of these, 33 (69%) migrated only one tier down. Assessing N-category, 49/169 (29%) migrated to a lower category, of whom 40 from N2 to N1. Only one patient migrated to a higher N-category, but with concomitant progression to M+ disease. The remaining 119 (70%) were judged to have a stable N-category at CT. An isolated change of T or N-category following NAC should not be interpreted as formal response or progression, as response evaluation in this study used the composite UICC TNM-stage.

**Table 1:** Clinical variables for patients with resectable gastric cancer 2007–2016 receiving neoadjuvant chemotherapy (NAC). Response was evaluated by TNM downstaging at repeated CT-scans, *n* = 169 (%)

	*Total*	*Response*	*Stable disease*	*Progression*	p value*
***Age category***					***0.55***
< 60 years	48 (28%)	19 (40%)	23 (48%)	6 (13%)	
60–70 years	76 (45%)	34 (45%)	38 (50%)	4 (5%)	
> 70 years	45 (27%)	20 (44%)	23 (51%)	2 (4%)	
***Gender***					***0.47***
Male	118 (70%)	54 (46%)	55 (47%)	9 (8%)	
Female	51 (30%)	19 (37%)	29 (57%)	3 (6%)	
***Tumour location***					**0.03**
Cardia	65 (38%)	32(49%)	29 (45%)	4 (6%)	
Corpus	32 (19%)	19 (59%)	12 (38%)	1 (3%)	
Antrum	51 (30%)	19 (37%)	30 (59%)	2 (4%)	
Diffuse	21 (12%)	3 (14%)	13 (62%)	5 (24%)	
Disease stage**					***< 0.01***
Stage 1b	14 (8%)	5 (36%)	9 (64%)	0	
Stage IIa/b	54 (32%)	15 (28%)	39 (72%)	0	
Stage IIIa	32 (19%)	18 (56%)	13 (41%)	1 (3%)	
Stage IIIb	42 (25%)	20 (48%)	17 (41%)	5 (12%)	
Stage IIIc	27 (16%)	15 (56%)	6 (22%)	6 (22%)	
T-stage**					***0.04***
T2	22 (13%)	11 (50%)	11 (50%)	0	
T3	33 (20%)	11 (33%)	22 (67%)	0	
T4a/b	114 (67%)	51 (45%)	51 (45%)	12 (11%)	
N-stage**					***< 0.01***
N0	53 (31%)	10 (19%)	41 (77%)	2 (4%)	
N1	50 (30%)	24 (48%)	24 (48%)	2 (4%)	
N2	51(30%)	33 (65%)	15 (29%)	3 (6%)	
N3	15(9%)	6 (40%)	4 (27%)	5 (33%)	
Lauren classification***					***0.79***
Diffuse	68 (40%)	26 (38%)	37 (54%)	5 (7%)	
Intestinal	73 (43%)	33 (45%)	34 (47%)	6 (8%)	
Mixed	27 (16%)	14 (52%)	12 (44%)	1 (4%)	
***Number of cycles delivered***					***0.41***
< 3	24 (14%)	12 (50%)	11 (46%)	1 (4%)	
3	145 (86%)	61 (42%)	73 (50%)	11 (8%)	

*Chi-square monovariable analysis

**Radiological staging at time of diagnosis (rTNM)

***One patient with unspecified Lauren category omitted

### Inter procedure reliability, CT stage after NAC (yrTNM) vs pathologic stage after surgery (ypTNM)

**Fig. 3:**
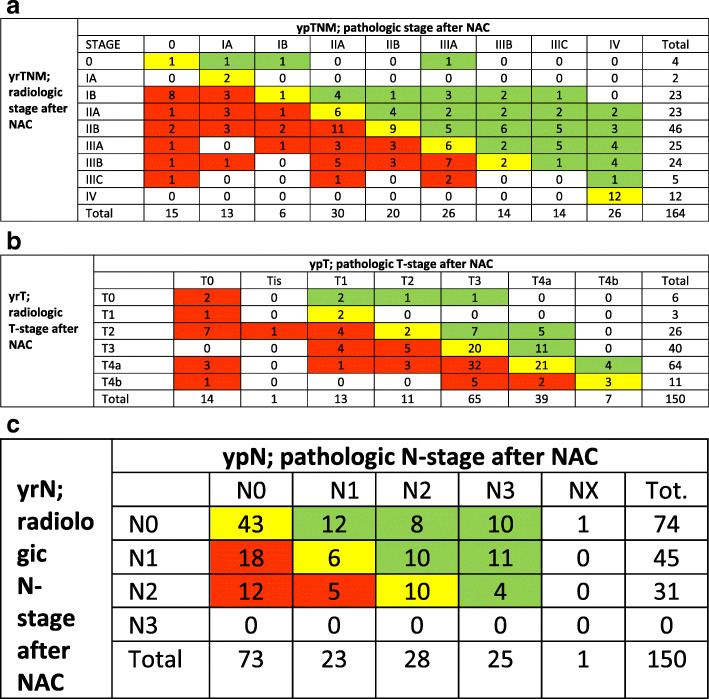
Correlation between radiologic and pathologic UICC TNM-stages **a**) CT-stage following NAC (yrTNM) vs definitive pathologic stage (ypTNM), *n* = 164. **b**) CT T-stage following NAC (yrT) vs definitive pathologic T-stage (ypT), *n* = 150. c) CT N-stage following NAC (yrN) vs definitive pathologic N-stage (ypN), *n*= 150. Green = understaging, yellow = concordant staging, red = overstaging

Of the 169 patients restaged by CT following NAC, 12 were diagnosed with M+, and an additional five with M0 were deemed unfit for surgery. A total of 153/169 (91%) were referred for surgery, including one patient diagnosed with M+ at CT evaluation. Perioperatively, 14 were diagnosed with carcinomatosis unrecognized at restaging CT, with 11of these receiving resection (Fig. 1). This implies a CT sensitivity of 46% and specificity of 100% in detecting peritoneal carcinomatosis. At histologic examination of resected specimen, one patient was further diagnosed with tumour cells in the omentum (M+), not acknowledged at CT or at operation. Of note, none of the operated patients had a locally non-resectable tumour. Altogether, 164 patients had an ypTNM-stage assigned to be compared to the yrTNM-stage. Only 39/164 (24%) had concordant stages, 38% overstaged and 38% understaged at CT, ƙ = 0.06 (95% CI 0.004–0.12). CT found complete radiologic response in four patients, compared to 15/164 (9%) with histologic complete response (Fig. 3a). Since 19/169 (11%) were not resected, the number of ypT- and ypN-stages was correspondingly lowered. For T-stage, CT was concordant in 50/150 (33%), overstaged 46% and understaged 21% (Fig. 3b), ƙ = 0.1 (95% CI 0.01–0.18). Overstaging by CT following NAC was particularly common, staging only six tumours as T0 compared to 14 histologic T0 cancers. For N stage, CT was concordant for 59/150 patients (39%), overstaging 23% and understaging 37% (Fig. 3c), ƙ = 0.097 (95% CI 0.02–0.18). Most cases of misclassification were due to understaging with no patients staged to yrN3, whereas the pathologic report assigned 25 patients to N3 status.

### The discriminating ability of radiologic response mode on long-term survival rates.

**Fig. 4:**
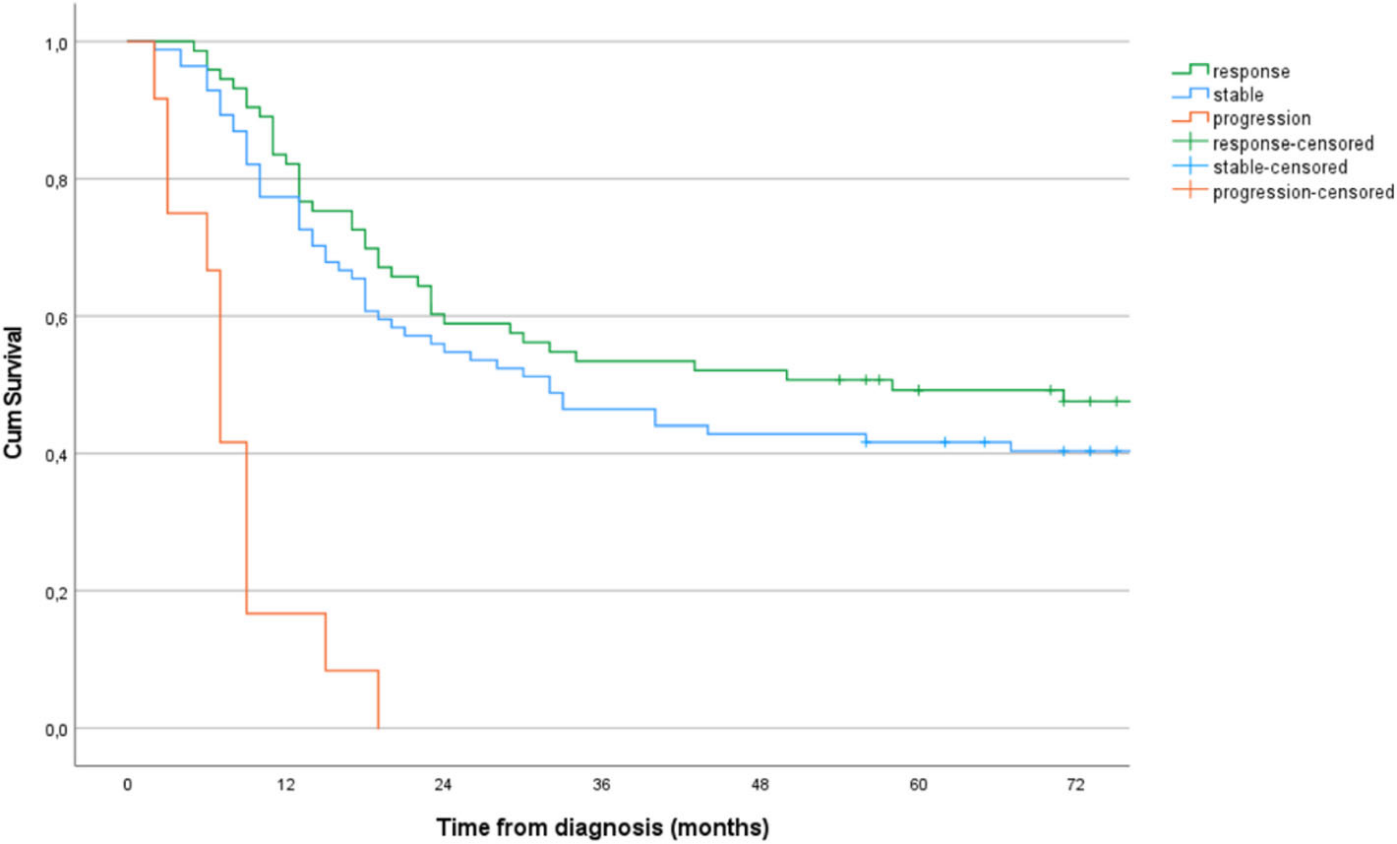
Kaplan–Meier plot of estimated overall survival according to response group. Response assigned by comparing radiologic stage following neoadjuvant chemotherapy (yrTNM) to radiologic stage at baseline (rTNM) for *n* = 169 patients with true gastric cancer

Long-term survival rates for all patients stratified on response mode are depicted in Fig. 4. As expected, patients with progressive disease at CT had a substantially inferior survival since all were allocated to this group due to metastatic disease at restaging CT. No significant difference was found in long-term survival rates for patients with stable disease compared to those judged to be responders at restaging CT, log rank *p* = 0.237 (Fig. 4).

## Discussion

Two principally different approaches for evaluating tumour response following NAC are available. Histologic criteria such as the Becker tumour regression grade [12] or the Mandard score [11, 19] both restricted to evaluate the resected specimen or radiologic methods as UICC-*downstaging* [18] or RECIST-*downsizing* [13] using metrics to tumour and lymph nodes. There is no consensus on preferred approach, and clinical implications of findings are still discussed [7].

An advantage of radiologic response is the possibility to include all patients receiving NAC and not by virtue exclude those not resected. Further, radiologic methods include N-stage and M-stage in the evaluation, with histopathologic methods restricted to evaluation of the main tumour [11, 12]. For *downsizing,* RECIST compares unidimensional measurements of target lesions before and after NAC, whereas non-target lesions are assessed as present or absent [13]. The 2009 revised version, based on data from more than 16 trials, had no gastric cancer patients included [20]. The gastric primary is counted as a non-target lesion, whereas lymph nodes are required to be ≥ 15 mm at diagnoses to be classified as a nodal target lesion. Applying this to the present study would leave only 18 patients with a nodal target lesion to measure (data not shown). Hence, potential advantages can be envisaged moving strategy from RECIST and *downsizing* to radiologic TNM *downstaging*. First, evaluation is not limited to metric measurements but is able to grasp a wider picture by including both T-, N- and M-categories, with the added value of using a common language for radiologic evaluation and pathologic examination of the specimen [21]. Second, long-term survival rates are prognosticated by UICC stages, and downstaging following NAC may intuitively be expected to translate into improved long-term survival rates. Tumour size alone or downsizing by metrics is less correlated to survival rates [22, 23].

The findings of this study, however, suggest that CT *downstaging* does not serve as an adequate tool in evaluating response to NAC. CT assigned stages following NAC are unreliable, and CT-response mode is unable to discriminate strata of significant different long-term survival rates. First, half of the patients developing carcinomatosis during NAC had the progression undetected at restaging CT. Second, any acknowledged progression was only to metastatic disease (M+), with no tumours deemed to have progression restricted to T-category. Reason suggests otherwise, as illustrated by comparing baseline rT-stage to ypT-stage, identifying 15 patients with tumours judged to migrate to a higher T-stage (data not shown). Along the same reasoning, CT following NAC classified only one patient progressing to a higher N-stage from baseline CT, whereas radiologic N-stage at baseline compared to pathologic N-stage suggested that 43 patients progressed to a higher N-stage (data not shown). Third, CT downstaging failed the objective of assigning patients to groups with different long-term survival rates based on response mode. As expected, patients progressing on NAC demonstrated inferior survival since the group was constituted by patients with metastatic disease at restaging CT, but survival rates for responders and patients with stable disease showed no significant difference (Fig. 4).

An Achilles heel of radiologic response evaluation is the need to accurately stage patients, both at baseline and following NAC. In a setting with upfront surgery, the radiologic concordance comparing baseline CT to pathologic stage lies within 69– 88% for T-stage [24–26] and 51–71% for N-stage [27], the latter further obscured by a lack of agreement on cut-off size for nodes to be deemed infiltrated [28]. Following NAC results deteriorate. Studies report a concordance between yrT and ypT ranging from 33–57% [24, 29, 30]. Chemotherapy-induced inflammation and oedema imped the evaluation of tumour depth invasion [31], and CT has a low ability to differentiate chemotherapy induced fibrosis from vital tumour tissue, leading to an overstaging of T-category [24]. For N-stages, concordance between yrN and ypN is reported down at 37–51% [24, 29, 30], which is unfortunate since pathologic lymph node status following NAC (ypN) is considered a main determinant for long-term survival in gastric cancer [32].

In the present paper, concordance between radiologic stage and histologic stage following NAC is in the lower range, for T-stages at 33%, for N-stages at 40%. However, even more informative is the correlation using the Cohen kappa statistic, although often not provided [24, 29, 30]. This quantification adjusts for concordance by chance and returns a lower but more realistic number on how the two methods of staging compare. By no doubt, response evaluation by CT *downstaging* is unreliable and to some extent overstates T-stage and understates N-stage by two or more tiers. Correlation as seen in this paper, with kappa statistics at or below 0.1 both for the UICC T-, N- and composite TNM-stages, is rated as very poor [33]. Of further note is a low sensitivity for CT to detect carcinomatosis [34]. In the present paper, restaging CT identified carcinomatosis in 12 of 169 patients, whereas an additional 14 patients had carcinomatosis at operation. All patients progressing to metastatic disease did so from advanced disease stages at the outset. As laparoscopy has a better sensitivity for detecting peritoneal metastases [34], guidelines today recommend this when facing advanced disease in a curative setting [1], further limiting the role of restaging CT in patients following NAC.

A further key point is that CT following NAC did not detect any deeply located parenchymal or extra-abdominal metastases that would have gone undetected by surgical exploration. Further, no patient was denied surgery following tumour progression to non-resectability, and none of the patients explored were found to have locally non-resectable tumours. Rather, the low accuracy of CT staging could misguide decision making and contribute unjustified to deny a patient surgery. In a frail patient with a large cardia tumour and lack of downstaging, risky surgery could be cancelled. However, as seen from Fig. 3b, some yrT3 or yrT4 cancers are found to be ypT0-2. These observations are in line with findings in a similar, recently published study from the Netherlands [35].

A strength of the present study is that it is population-based with no selection bias. Multiple factors may, however, affect the quality and accuracy of the CT response evaluation. It is considered favourable that all CT examinations were performed at the same institution, using a standardized protocol. CT slice thickness was set at 1.5–3 mm, whereas other studies often report slice thickness of > 5–7 mm, which is not up to standards of current CT- technology [25, 36]. A limitation of the study is its retrospective nature and that a single gastro-radiologist performed the radiological response evaluation. Although securing a consistent evaluation of the CT scans, assessing inter-observer agreement is rendered impossible.

## Conclusion

No consensus exists on how to perform response evaluation following neoadjuvant chemotherapy (NAC) for upfront resectable gastric cancer. The RECIST criteria using unidimensional measures of target lesions are not validated for gastric cancer. Based on data from a 10-year experience, we conclude that CT-scan with radiologic UICC restaging following NAC is not suited for response evaluation either. Accuracy of such staging compared to final pathologic stage is poor and radiologic d*ownstaging* as measure of response evaluation unreliable. Further, CT following NAC had a limited value in surgical decision making as no patient developed locally non-resectable tumour or deep organ metastases and selective use of diagnostic laparoscopy in patients with advanced localized disease at the outset will better identify patients progressing to peritoneal carcinomatosis.

## Data Availability

The datasets generated and analysed during the current study are not publicly available due to hospital policy but are available from the corresponding author on reasonable request.

## Abbreviations

NAC: Neoadjuvant chemotherapy
UICC: International Union Against Cancer
AJCC: American Joint Committee on Cancer
TNM: Tumour (T), nodes (N), and metastases (M)
rTMN: Radiologic TNM at diagnosis
yrTNM: Radiologic TNM after neoadjuvant treatment
ypTNM: Pathologic TNM after neoadjuvant treatment
RECIST: Response Evaluation Criteria in Solid Tumours
NCR: Norwegian Cancer Registry (NCR)
NPR: The Norwegian Patient Register
EPJ: Individual electronic patient journals
